# Infantile Convulsions

**Published:** 1867-01

**Authors:** A. P. Merrill


*Infantile Convulsions.* By A. P. Merrill, M. D.

In city necrology, the term "infantile convulsions" is generally made to include all the convulsive diseases of children that may be reported, whether they appear in the form of trismur or *tetanus nascentium*, or other kinds of tonic and clonic spasms, ending in destruction of life. The disease, in one form or other, prevails extensively, at all times and in all places, throughout the habitable globe, causing great mortality.

The causes which have been assigned to it are so many and various that it may well be doubted whether its etiology is better understood now than at first. Much has been written of its pathology, also, but with such uncertain aim that scarcely any two authors agree, except in regard to the fact that a certain disorder of the nerves exists, producing more or less vascular congestion. The treatment of the disease has been so varied, and remedies have been so multiplied, with such small influence over results, that grave doubts are entertained by many as to the efficacy of medical treatment of any kind; and we even find prominent physicians claiming, as the result of their experience, that it is often advisable to intrust the suffering victim to the curative powers of nature alone; and contending, especially in cases affecting very young infants, that all known remedies are wholly ineffectual. In more advanced childhood spontaneous recoveries more frequently take place, and the disease is often cured by active medication; but the new-born babe is doomed by convulsive disease to almost certain death. So well is this understood that in many cases a physician is not called in, and remedies of any kind are only applied sparingly and without skill.

In regard to causes, we have been strangely told of certain displacements of cranial bones, as if in distrust of nature in preparing the head for the pressure of birth, and for recovery afterwards; and quite as strangely of the careless excision of the cord beyond the ligature, where cutting or tearing could have no influence over the point of junction with the body; and we have been told of exposure to cold, of filthy habits of life, of hereditary taints, of indigestible food, constipation, diarrhœa, worms, and teething; and a variety of remedies have been devised for these particular exigencies, but the death-rate is not decreased. In all large cities asylums are provided for poor and homeless infants, nurses are duly trained and instructed, physicians of eminence devote their time to the treatment, the materia medica is explored for remedies, nostrums are invented and applied without limit, all phases of charlatanry claim the confidence of the public, and still the death-rate remains the same. Convulsions continue to decimate the human race in early childhood, and to people the cemeteries with infant dead.

Like many other fatal diseases, infantile convulsions produce the largest mortality in hot climates and in marshy districts. This, coupled with the fact that in many cases the periodic movement is undoubted, and with the equally important fact that whenever an intermission is secured, antiperiodic remedies are most efficacious in affording relief, affords us good reason for believing that these convulsive diseases are of a periodic character, and due to malarial influences. But the greatest mortality is with new-born babes, sometimes attacked as early as the second day after birth, and these rarely survive the first convulsion. With them, therefore, there is no means to determine the question of periodicity, for there is no reaction, no intermission, and no return of the disease as in intermittent fever. It may be supposed, too, that the period of exposure and of incubation are in such cases too short. Yet it is by no means improbable that the exciting cause may have reached the infant through the medium of the mother, or that under hereditary influences the predisposition of the child may have been such as to reduce the incubative period to a single day. Be this as it may, there is abundant evidence that the mother often suffers from periodic fever, while the infant is suffering with convulsions, and antiperiodic medicines have been found remedial and prophylactic for both.

In the treatment of infantile convulsions, I have known physicians to depend mainly upon quinine, given generally by enema, during the continuance of the spasms; but the more common practice is to give emetics of ipecacuanha, and to apply mustard and hot bathing, with stimulating and purgative enemata. Mustard is a powerful remedy in this and other congestive diseases. I have used it with satisfaction in baths, by sinapisms, by enemata, and by the stomach. But all these means are inferior to blood-letting in giving relief to the spasms. Great care and skill are required, however, in bleeding young children in congestion from chill, lest they sink under the operation and die from exhaustion. Blood must be drawn in such cases in small quantity at a time, and the operation must be repeated as recuperation takes place, and the child can bear it. It is prudent, too, to conduct the depletion under pretty active stimulation. In proportion to the severity of the chill and the congestion accompanying it, is the danger of prostration from the sudden loss of blood; but in the same proportion is the necessity for the remedy, and its efficacy, when properly applied. Reaction is not only secured and convulsions relieved, but the resulting febrile exacerbation is less violent than under the other treatment above referred to, and the subsequent intermission is more complete.

In the selection of cathartic remedies I have found calomel to be the safest and the most efficient, not only in this, but also in most other diseases of children, and the younger the child the more it is to be preferred over other remedies of this class. An infant may take a quarter to a half grain of calomel the day it is born, with less risk of injurious effects than from any other cathartic. Opium, on the contrary, which is so frequently resorted to, I consider an exceedingly dangerous medicine for infants, and especially when they are very young. The cordials and soothing syrups in such general use depend upon opium in some form for their composing effects, and frequently they do compose and soothe the suffering babe, to the great relief and satisfaction of nurses, but to the injury of the child, sometimes even to the destruction of life. The healthy action of the digestive organs so necessary to the health of all persons, is especially so to infants, and nothing so readily deranges infantile digestion as opium.

From all the teachings of experience in regard to this matter, there scarcely seems to be room to doubt that infantile convulsions, which are so prevalent and destructive to life, are, when not dependent upon organic lesion, or trumatic irritation, mostly of a periodic character, and produced by the congestions consequent upon the cold stage of fever. In the adult subject the tendency of these congestions to produce convulsions is very strong; and in the puerperal state, in yellow fever, and in the graver forms of bilious remittents, general convulsions are not an uncommon concomitant of the initiatory chill. Females are more liable to febrile convulsions than males. Children of three to five years of age are more subject to them than adults; and it is rarely the case that a child less than a year old suffers with any considerable severity without more or less of spasmodic action, showing that the predisposition to convulsions is in some proportion to the degree of nervous irritability. In young infants the premonitory signs of an attact are crying and refusal of food, followed by coldness and a purple hue of the hands and feet, with clenching of the fingers and toes.

It may be objected that the persistency of spasmodic action in young infants is evidence against the existence of periodicity; and it may be contended that the subsidence of the chill should be followed by an intermission of the convulsion. But it must be considered that spasm once produced by congestion of the nerve centres, or of the larger blood-vessels, as the result of disorderd innervations, soon becomes habitual, and may continue after the primary conditions producing it are measurably relieved; or, it may well be supposed that the relief of congestion on subsidence of chill may be only partial, still continuing to exist to such extent as may be necessary to keep up spasmodic action, just as many symptoms of chill in the adult subject often remain after reaction is established. Without entire relief of the abnormal nervous action, the irritable muscles will continue liable to convulsive movements until the vital powers are exhausted, or the convulsions will intermit for short periods of time from temporary exhaustion, to recur again with increased energy as cerebral and nervous power are partially restored by comparative rest. The disease, in fact, looses its periodic character in the intensity of the morbid lesions produced by it, and becomes a disease of local congestions and inflammations, with convulsive manifestations as their effects, and as evidences of their existence.

The most effective remedy yet discovered for convulsions arising from congestion is chloroform, used exclusively as an internal remedy. Its full physiological effect, as evidenced by sleep, is certainly remedial of that disordered innervation which causes congestion and chill. Whether the disease attack the child during the first week or month, and is called trismus or tetanus, or seize upon older children in the form of tonic or clonic spasm, chloroform internally, in proper doses, always affords the best chance of relief; and when relief is thus obtained the paroxysm is not likely to return. Preventive measures should not, however, be neglected, especially in malarial districts. Quinine should be given to both mother and child, and, if practicable, there should be a change of residence to avoid the influence of malaria, for it is difficult to guard against a return of the disease, especially at septenary periods, while the cause is constantly acting.

Chloroform is best given to young children in milk; but if the spasm is severe and persistent, deglutition is proportionally difficult, and in that case the remedy is poured gradually into the mouth without the admixture of vehicle, taking care that it does not spread over the skin of the chin and neck. The mucous membranes suffer no injury from this contact of pure chloroform, beyond the very temporary effect of the stimulation; but the skin sometimes is vesicated with subsequent ulceration. I have given to infants within the month from one to five drops every fifteen or twenty minutes until the spasms were relieved and sleep secured. For older children the dose may be increased in proportion to the age and the vigor of constitution, and be repeated according to the intensity of the disease. I have given a teaspoonful to a child three years old, undiluted, with the happiest effect; and to one five years old I have given the same quantity, and for want of entire relief repeated the dose in twelve minutes with success. This was a case of severe and long continued convulsions, with great prostration of vital energy, which seemed to require such quick repetition of the dose lest death might ensue. The child slept and recovered.

In my treatment of various forms of disease by the internal use of chloroform, given in hypnotic doses, it has sometimes happened that the patient has afterwards enjoyed much better health than previously. This, taken in connection with the fact that chloroform is powerfully destructive of insect and animalcular life, and in connection with the success of the remedy as a vermifuge, affords reason to suppose that one of the important benefits to be derived from such treatment is to rid the system of worms, and of every kind of animalcule and morbific germ, whether existing in the cavities or the solids and fluids of the body; for the stomach and bowels cannot be charged for any length of time with the vapor of chloroform, into which the doses given are very soon converted, without pervading the whole circulatory system, and making itself manifest in every secretion and excretion of the body. No extraneous vital organism can, by any possibility, escape its destructive influence. This gives to the internal exhibition of chloroform a range of usefulness far exceeding that to be derived from its remarkable efficacy in disordered innervation merely, and makes it the most valuable of all the remedial agents yet known to man. Indeed, when we consider the large proportion of fatal diseases, both to men and animals, which are supposed to be attributable to the influence of entozoa and various kinds of animalcula, this feature in the internal use of chloroform possesses a value which can scarcely be overestimated.—*Med. Record.*

